# How Will the War Now Raging Be of Benefit to Mankind?

**Published:** 1916-03

**Authors:** F. Paschal

**Affiliations:** San Antonio, Texas


### TEXAS MEDICAL JOURNAL

Dr. F. E. Daniel, Founder. Established July, 1885

MRS. F. E. DANIEL, - Publisher and Managing Edito

Published Monthly.—Subscription, $1.00 a Year.

VOL. XXXI AUSTIN, MARCH, 1916 No. 9

The publisher is not responsible for views of contributors

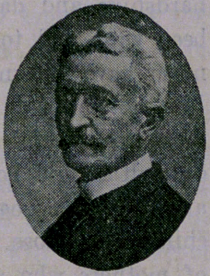

### Original Articles.

### How Will the War Now Raging Be of Benefit to Mankind?

BY F. PASCHAL, M. D., F. A. C. S., SAN ANTONIO, TEXAS.

It is just as well to be understood in the beginning that I am not interested in the diabolical inventions for the destruction of life, but I am intensely interested in the improvements that this war has wrought, and will continue to bring forth, for the salvation of life. It is impossible to even think, without the deepest regret, of the terrible loss of life that has taken place, and which will continue, as long as the warring nations of Europe do not cease their struggling for supremacy. Undoubtedly it is the earnest wish of every neutral that the war may soon end. But, however much the war is regretted, the fact remains that the experience gained will be of untold benefit to the entire civilized world. Already the lessons that the medical profession has learned are invaluable to humanity. The prevention of typhoid and typhus fever, the control of cholera and tetanus, the possibility of living in underground trenches for months and making such abodes sanitary, are great triumphs. It probably will never be said that preventable diseases killed more men in this war than were killed by powder and lead, as was the case in previous wars. The splendid hospital equipments, the facilities for collecting the wounded and conveying them to hospitals, the care of the sick and the treatment of wounds and many other important matters have marked an era of advancement in military surgery, military medicine, and military hygiene that is simply marvelous.

A thing of interest is to consider the possible effect that the war will have upon the offspring of the soldiers that survive it.

The fathers of millions of children will have endured the severest hardships and dangers, developed heroic courage, and will have been subjected to thorough disciplining. Probably millions may never have known what military training was prior to enlistment. Is it not probable that the children of the survivors will be braver, more resourceful and more independent and better able to meet difficulties and contend successfully with the problems that will arise in after years in their struggles for an existence, than the children of those who were undisciplined? There will be millions of mothers who also will have experienced hardships, trials and privations, and on whom necessity forced self-reliance and helped to develop latent abilities that perhaps would not otherwise have been developed. Is it not likely that children born of such parents will be better disciplined because of the training misfortune brought to these parents? Is it not reasonable to suppose that fathers and mothers having been subjected to strict discipline and knowing its value will apply it in rearing their children? No doubt many children will be greatly improved, for the reasons above stated. If this should prove to be the case, and it is almost certain that it will, it will be a blessing to future generations of the warring and other nations. The majority of children "grow up" undisciplined, unambitious, and as a result homes and countries suffer.

The acquisition of knowledge is, of course, always beneficial, and its dissemination is usually by contact brought about through amalgamation and association. Emigration will be large after the struggle is ended. It is a notorious fact that frequently failures in life are due to the lack of ambition and advancement, to a large degree caused by the parents' ignorance of the necessity of home discipline.

It is true that a certain percentage of men and women will develop different forms of nervous diseases on account of the intense nervous strain and suffering that they will have been subjected to, but the number will be small compared to the number of those who will be improved in stability and mental development as the result of the war.

The world need never fear that the old and decrepit men alone will survive to perpetuate the races of the contending countries—there will be millions of young men spared to be fathers of noble people and the experience and training that they will have received will make them braver, stronger and more self-reliant than they perhaps were before the war. They will be better able to cope with economic conditions, and consequently be more intelligent and active in rebuilding the ruined fortunes of their lands. With such men for conservators it will not be long before the nations will recover from the staggering blows they will have received.

It is true that enormous debts will be put upon the present and future generations, but these debts will be only incentives for greater activities for their cancellation. The arts, sciences, and industries will take on new life and the land will be made to produce more, and the countries that are war-torn will recover from its effects more rapidly than the world can realize.

Regret of the war will never cease to be, but the awakening and rapid advancement and the lessons taught will ultimately be of benefit to mankind. Some of the benefits will be immediate, and in many cases are already felt, others will be delayed, but finally they will be acknowledged.

I am not an advocate of war, and pray that this one will stop and that the world may never have another, but wars are just as sure to occur as men are to live, and to expect universal and everlasting peace is just as absurd as to expect the sun to cease shining. Considering this war from a strictly scientific point, it must be admitted that it has largely taught, and will continue to teach, much that is very valuable to the present and much that will be of value to future generations. It is horrifying in the extreme to think that benefits should be the outcome of immense suffering and loss of life. But the responsibility for war is upon those who could but would not prevent it. And statesmen of the present and future will have been taught the necessity for toleration and charity; but the honor and dignity of their countries will be considered first above all things else.

Every calamity, deplorable as it may be, usually is followed by profit by the experience gained and applied in the prevention of other and greater calamities.

Viewed strictly in an unbiased manner, one must admit that with all of its horrors and at the cost of millions of lives, suffering, anguish, bereavement and ruined countries, that finally benefit will accrue. This especially applies to military medicines, surgery and hygiene, and the knowledge obtained in these lines will also be used for the benefit of the public health. Mistakes will be profited by, and the weak points made strong; and in all likelihood a stronger, more intelligent and energetic people will be developed to benefit the world.

